# Bibliographic dataset of literature for analysing global trends and progress of the machine learning paradigm in space weather research

**DOI:** 10.1016/j.dib.2023.109667

**Published:** 2023-10-18

**Authors:** Nur Dalila K．A．, Mohamad Huzaimy Jusoh, Syamsiah Mashohor, Aduwati Sali, Akimasa Yoshikawa, Nurhani Kasuan, Mohd Helmy Hashim, Muhammad Asraf Hairuddin

**Affiliations:** a Department of Computer and Communication Systems, Faculty of Engineering, Universiti Putra Malaysia, 43400 Serdang, Selangor, Malaysia; b College of Engineering, Universiti Teknologi MARA Johor Branch, 81750 Masai Johor Malaysia; c School of Electrical Engineering, College of Engineering, Universiti Teknologi MARA, 40450 Shah Alam, Selangor Malaysia; d WiPNET Department of Computer and Communication Systems, Universiti Putra Malaysia, 43400 Serdang Selangor, Malaysia; e International Research Center for Space and Planetary Environmental Science (i-SPES), Kyushu University, 819-0395 Fukuoka, Japan; f Department of Earth and Planetary Sciences, Kyushu University, 819-0395 Fukuoka, Japan; g Exploration and Space Science Division, Malaysian Space Agency (MYSA), 42700 Banting Selangor, Malaysia

**Keywords:** Bibliometric evaluation, Development trends analysis, Literature review data, Open-source R-package, Visualisation

## Abstract

The field of space weather research has witnessed growing interest in the use of machine learning techniques. This could be attributed to the increasing accessibility of data, which has created a high demand for investigating scientific phenomena using data-driven methods. The dataset, which is based on bibliographic records from the Web of Science (WoS) and Scopus, was compiled over the last several decades and discusses multidisciplinary trends in this topic while revealing significant advances in current knowledge. It provides a comprehensive examination of trends in publication characteristics, with a focus on publications, document sources, authors, affiliations, and frequent word analysis as bibliometric indicators, all of which were analysed using the Biblioshiny application on the web. This dataset serves as the document profile metrics for emphasising the breadth and progress of current and previous studies, providing useful insights into hotspots for projection research subjects and influential entities that can be identified for future research.

Specifications Table

**Table:** 

Subject	Earth and Planetary Sciences, Library and Information Science
Specific subject area	Bibliometrics/Scientometrics
Data format	Raw Filtered Analysed
Type of data	Excel files (dataset with numbers and labels) Table (dataset with numbers and labels) Figure
Data collection	Initially, 481 search queries were returned from the database acquired from Scopus and Web of Science (WoS). It returned search results which were retrieved on 21st August 2023. The final selection of the data follows the Preferred Reporting Items for Systematic reviews and Meta-Analyses (PRISMA) guideline protocol and adheres to inclusion and exclusion criteria to filter out irrelevant documents of which 207 articles were excluded owing to post-screening as results from articles duplication and out-of-scope, in a total of 274 eligible documents, which led to the bibliometric analysis.
Data source location	Acquisition process and processing were conducted in the following details: Institution: Universiti Teknologi MARA Johor Branch Country: Malaysia Electronic database: Web of science (https://www.webofscience.com) and Scopus (https://www.scopus.com/home.uri)
Data accessibility	Data are with the article. Repository name: Mendeley Data Data identification number: 10.17632/fjpwfwfcg9.2 Direct URL to data repository is available at: https://data.mendeley.com/datasets/fjpwfwfcg9/2

## 1 Value of the Data

• Compilation of research related to the progress and impact of machine learning in the field of space weather studies based on bibliographic records conducted from 2008 to 2023 (within 16 years).
• Scholars, researchers, scientists, policymakers, and agency stakeholders would benefit from the analytical information derived from the dataset, scrutinizing bibliometric outcomes, as it would enable them to better understand the specific area of research and its potential expansion.
• The reuse of the dataset would provide insights into analyses of scholarly publications, research productivity, changes in research activity over time, and future research directions, and may provide policy guidance that encourages collaborative work in multidisciplinary research.
• Methodological data analysis in analysing bibliographic records, which was accompanied by graphical visualisation in the form of graphs and charts, could be replicated for other bibliometric or scientometric studies.
• The findings of this study can be used to provide a broader perspective of this area, the development and growth of the research domain, its evolution, and potential future research areas.

## 2 Objective

Bibliometric indicators are a valuable approach for evaluating and analysing research literature in order to examine and investigate various themes and disciplines by examining production patterns, trends, and the impact of publications [1,2]. The primary aim of this article is threefold: to employ a methodological approach based on the PRISMA framework for conducting a literature review, to produce bibliometric datasets that specifically pertain to the topic of machine learning within the field of space weather and finally, to present these datasets in the form of graphical visualisations. The findings from this research paper constitute a starting point as an initial foundation for delving into exploring further research and conducting content analysis regarding the applications of machine learning in different sub-areas of space weather studies.

## 3 Data Description

**Fig. 1:**
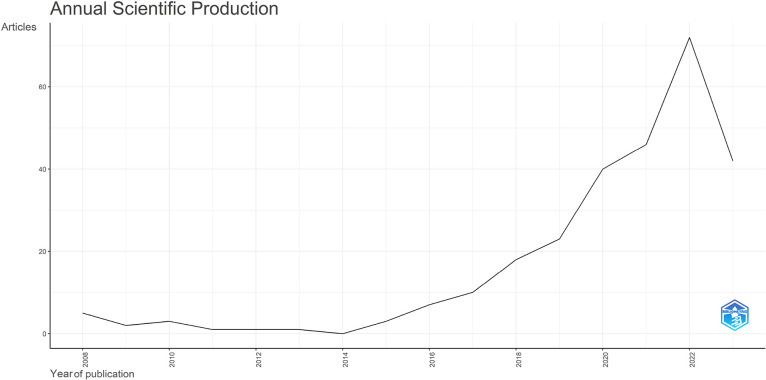
Annual scientific productions.

**Fig. 2:**
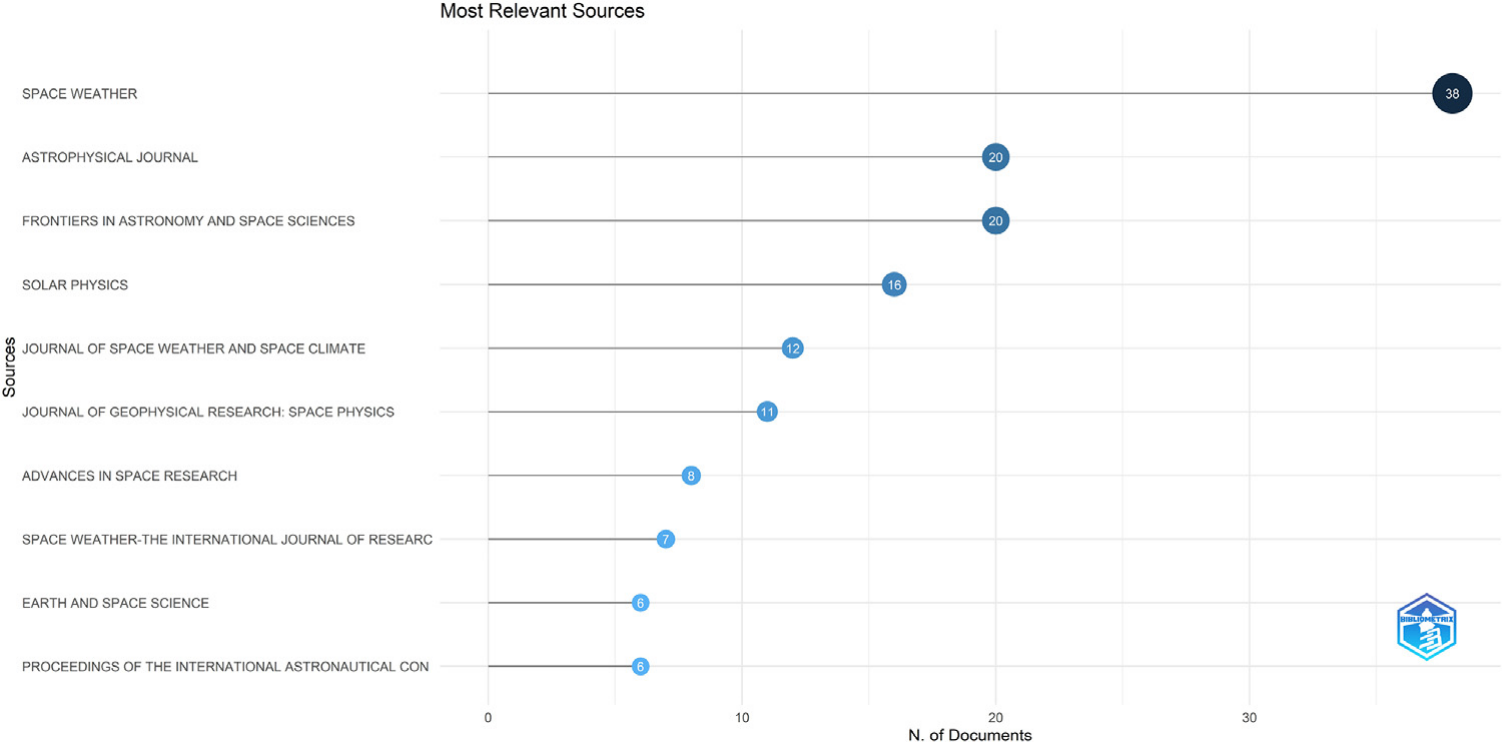
Most relevant sources.

**Fig. 3:**
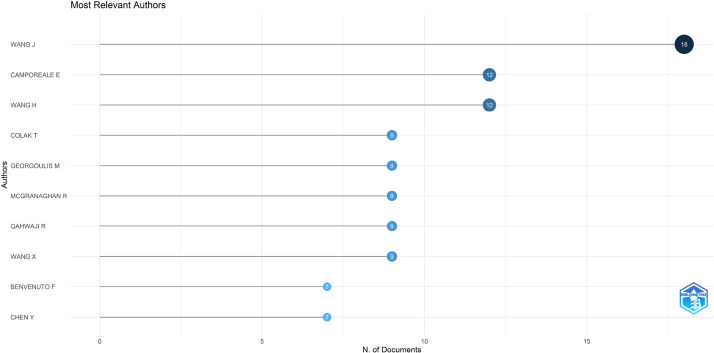
Most relevant authors.

**Fig. 4:**
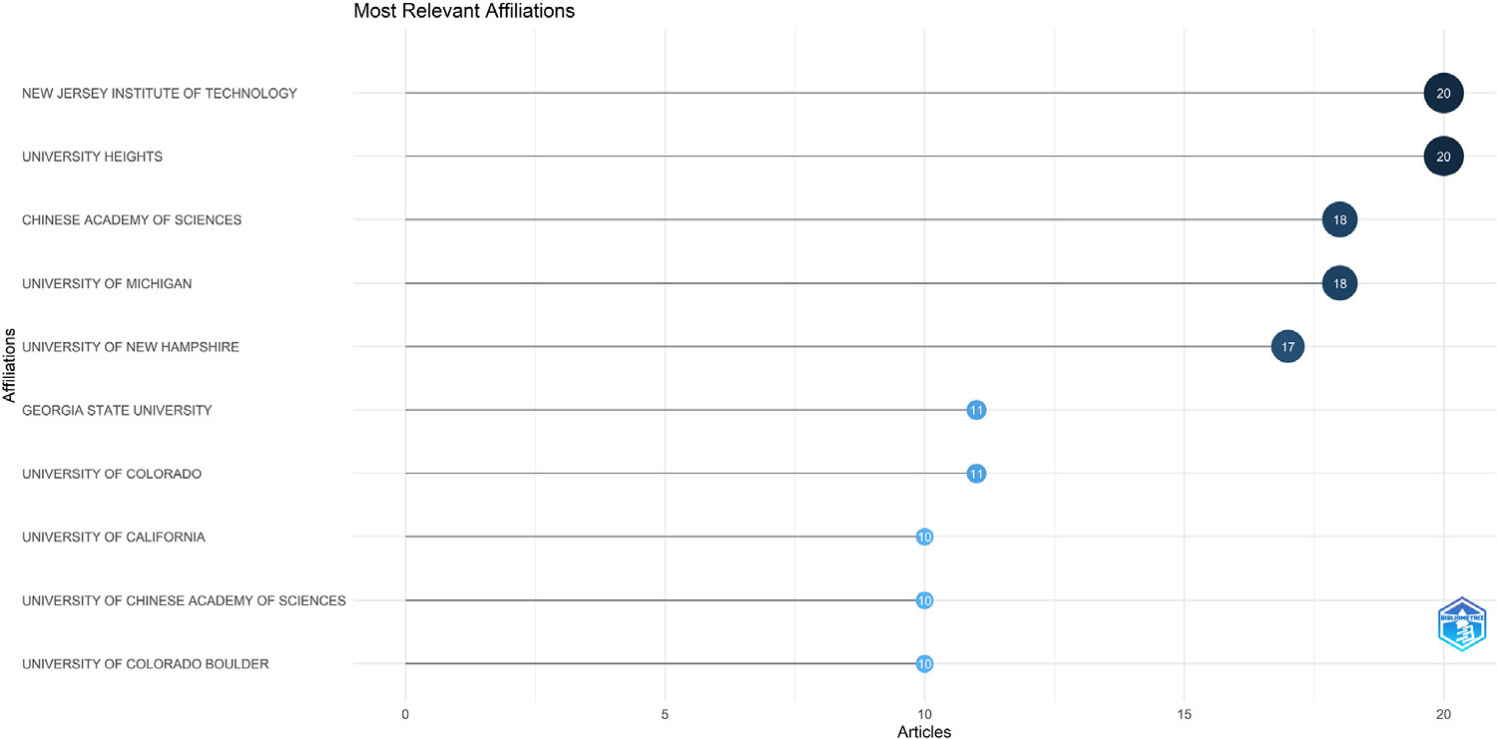
Most relevant affiliations.

**Fig. 5:**
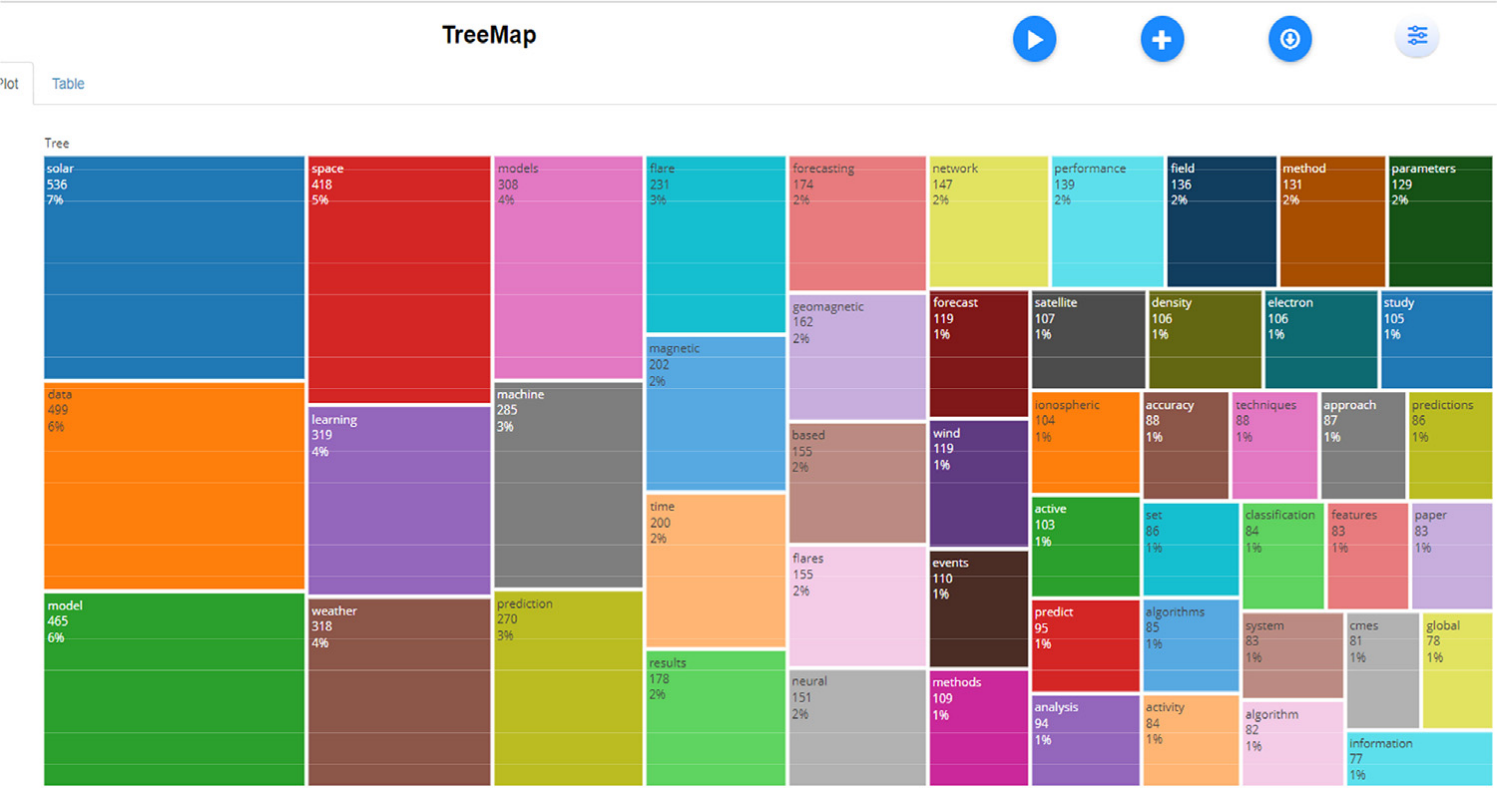
Most frequent words in abstracts.

**Fig. 6:**
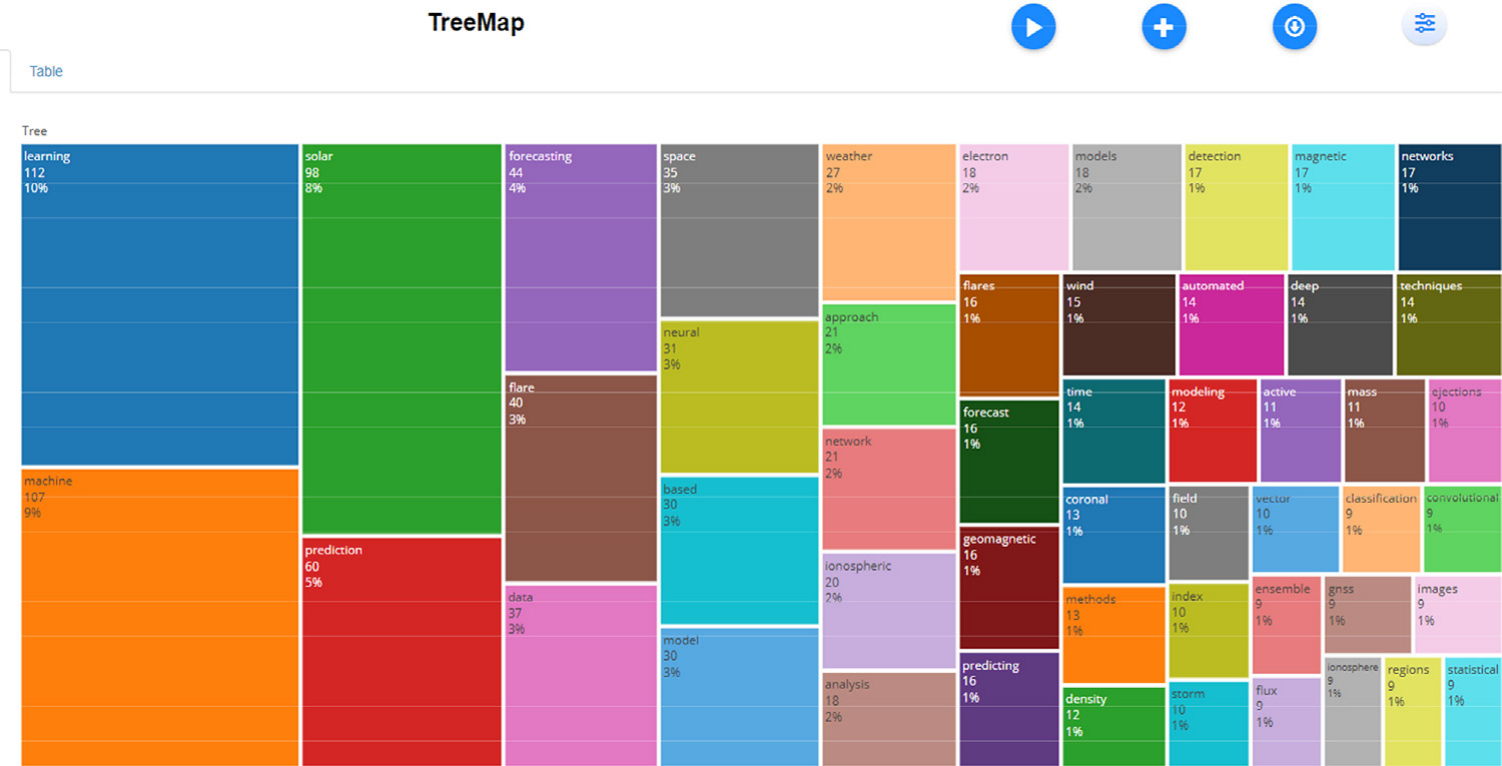
Most frequent words in titles.

The dataset contained bibliographic data related to machine learning and space weather studies. It is structured into three folders: Dataset, Analysed data_figures, and Analysed data_tables. Bibliometric analyses of publications, journal sources, authors, affiliations, and the most frequently reported words in the abstracts and titles are reported in this study. Fig. 1, Fig. 2, Fig. 3, Fig. 4, Fig. 5, Fig. 6 show annual scientific productions, most relevant sources, most relevant authors, most relevant affiliations, most frequent words in the abstract, and most frequent words in the title. Each of the figures accompanied by tables is provided in the folder of Analysed data_tables. All analyses, based on 274 documents, covering the years from 2008 to 2023, were demonstrated on included_dataset to be analysed with the Biblioshiny web application. To familiarise itself with the bibliographic dataset, the nomenclature of each metadata column in the dataset is as follows: AU-Author, C1-Affiliation, JI-ISO Source Abbreviation, AB-Abstract, DI-DOI, BE-Editors, BN-ISBN, SN-ISSN, SO-Journal, TC-Total Citation, PN-Part Number, PP-Publication Page,DB-Database, TI-Title, VL-Volume, PY-Publication Year, J9-29-Character Source Abbreviation, CR-Cited References, AU_UN-Author's Affiliation (disambiguated), SR_FULL- Short Full-Reference, SR- Short Reference.

## 4 Experimental Design, Materials and Methods

**Fig. 7:**
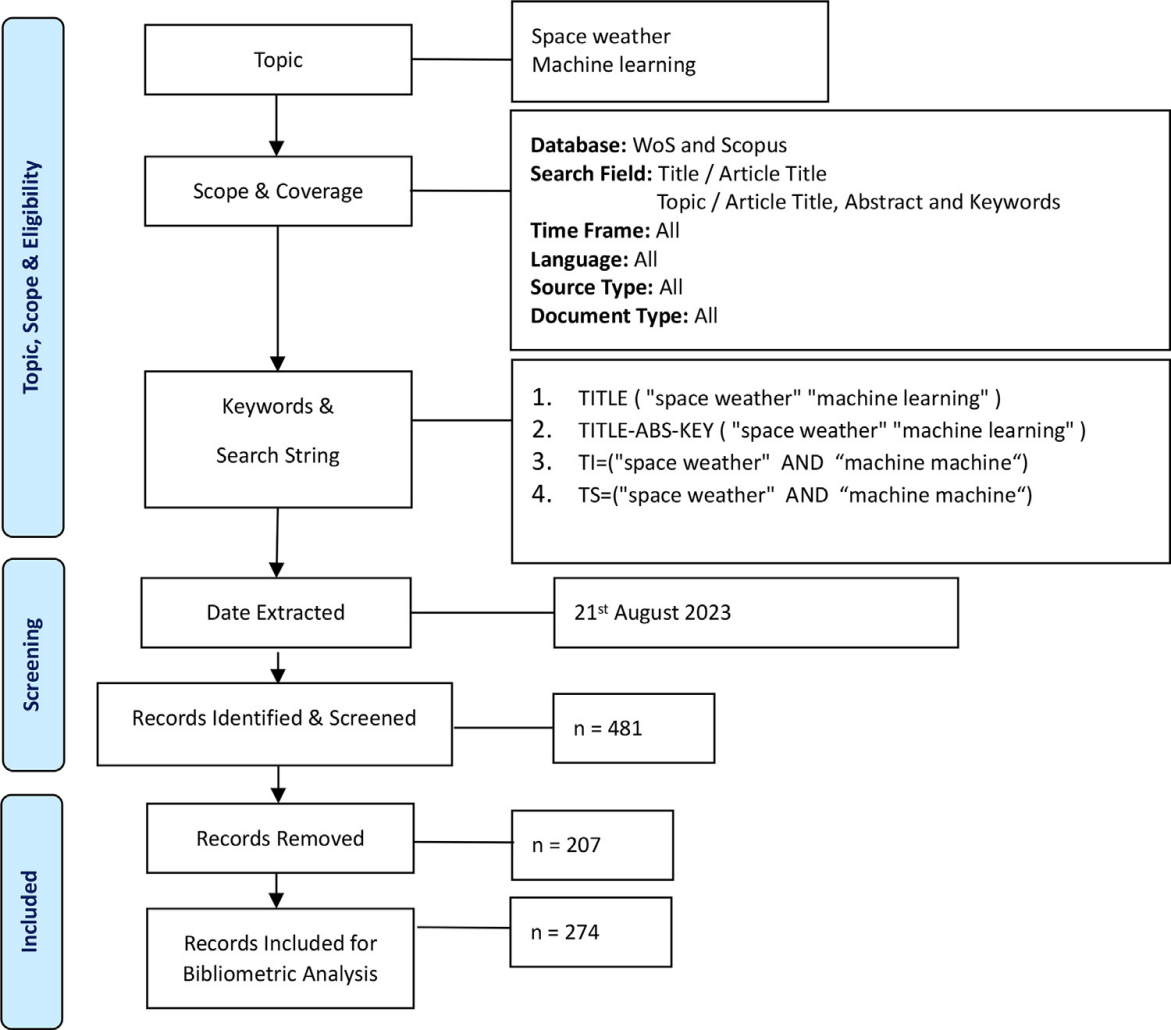
Flow diagram of the search strategy methodology [4,5].

As shown in Fig. 7, a systematic search strategy and framework are outlined to demonstrate the implemented procedures. The process involved in the bibliometric analysis consists of three (3) main elements. The first is to identify the topic and compile a series of literature; the second is to conduct a screening process; and finally, the selection of appropriate literature and bibliometric analysis. According to the PRISMA guideline process, the process of retrieving articles within the context of machine learning incorporates both reputable and wide-coverage databases retrieved from the Web of Science (WoS) and Scopus. The first database used in WoS is comprised of robust, high-quality assurance, reliable journals, and wide discipline coverage, while the second database selected in Scopus comprises the world's largest and most diverse existing literature. The combination of these two databases ensures quality assurance by including only reputable and robust journals to maintain the quality of the review process outcomes that have been conducted.

1. Topic, scope and eligibility
  The identification process in the systematic review revealed four key elements, comprised of the keyword search process and several search result options. Comprehensive keyword selection, as well as the resulting search articles, were executed and selected using meticulous query strings, which were also confirmed by a dominant expert in this field. Two searches were conducted to fully utilise the TITLE-ABS-KEY and TITLE in both databases. The search keywords were limited to two main keywords–machine learning and space weather–to enable the study to represent the core fields of the domain of the field of interest and obtain reasonable search results. Finally, overlapping documents were removed based on title and abstract screening to avoid redundancy between publications.
2. Screening
  The screening process involved selecting eligible articles and removing redundant documents. Initially, 256 search queries from the Scopus database and 225 results from WoS were returned to comprise return search queries with an additional recommendation of search results (August 21, 2023) with full records of retrieved research articles without filtering the search results. These two databases covered the time span of all years (2008–August 2023), and no filter options were selected to capture all metadata from both databases. To proceed with the bibliometric analysis, owing to the constraint of the matching filter option between the two datasets mentioned. The criteria of literature type, language, timeline, and country were all retrieved and collected from the database without filtering the options available to proceed with bibliometric analysis. The exclusion of some literature was conducted to clean concurrent documents and avoid filtering out irrelevant documents by examining them twice.
3. Included
  The post-screened articles were further analysed within the context of the intended studies by responding to the title and abstract. The author analysed 481 eligible documents; 207 articles were excluded due to post-screening that revealed article duplication and out-of-scope articles from the research domain of space weather and machine learning studies, resulting in bibliometric analysis using 274 included documents, which were evaluated with the Biblioshiny [3] web-based package application, which is useful for bibliometric analysis, citation metric analysis, and network mapping.

## Limitations

Not applicable.

## Ethics Statement

Authors have read and follow the ethical requirements for publication in Data in Brief and confirming that the current work does not involve human subjects, animal experiments, or any data collected from social media platforms.

## CRediT authorship contribution statement

**Nur Dalila K．A．:** Conceptualization. **Mohamad Huzaimy Jusoh:** Supervision, Writing – review & editing, Writing – original draft. **Syamsiah Mashohor:** Supervision, Writing – review & editing. **Aduwati Sali:** Supervision, Writing – review & editing. **Akimasa Yoshikawa:** Resources, Validation, Writing – review & editing. **Nurhani Kasuan:** Resources, Validation, Writing – review & editing. **Mohd Helmy Hashim:** Investigation, Writing – review & editing. **Muhammad Asraf Hairuddin:** Software, Validation, Writing – review & editing.

## Data Availability

Bibliographic dataset and analysis of machine learning in space weather (Original data) (Mendeley).
